# Mad Doggery

**Published:** 1861-09

**Authors:** 


**MAD-DOGGERY.**—European statisticians have said that as many persons die of hydrophobia in winter as in summer. Persons frequently become hydrophobic after having been bitten by dogs who were supposed by their owners to have been perfectly sound and well. In many cases the animal is killed instantly on having bitten a person, which person sometimes became hydrophobic, and sometimes not; sometimes within a month, at others not until two years have passed away. See JOURNAL OF HEALTH for July, 1857, where a man had been bitten by a dog nine months before, and had forgotten all about it, until reminded of it accidentally on Tuesday, May 26: on Friday following he died in horrible agonies from hydrophobia. From these statements important practical inferences should be drawn: First, never allude to hydrophobia in the presence of one who has been bitten by a dog; second, if bitten by any dog, at any season of the year, under any circumstances, whether the skin be penetrated or imperceptibly grazed by the animal's tooth, let the part be instantly sucked for one hour by one or a succession of persons, who are most perfectly certain that there is not the slightest sore or abrasion any where about the lips or tongue. Meanwhile, administer an enema, wash the parts freely in spirits of hartshorn every half-hour for three hours, and then every hour for the remainder of the day, putting fresh hartshorn in a clean saucer on each occasion; if any thing at all is eaten, let it be of the lightest, simplest kind. The object of the hartshorn, which is the strongest alkali, is to neutralize the poison, which, like almost, if not all, bites, is acid. One of the objects in eating but little, is not to use the strength of the system in digesting the food, but rather let it be employed in repelling diseased influences. In the mean time, send for a physician.

